# Phase 1 Human Immunodeficiency Virus (HIV) Vaccine Trial to Evaluate the Safety and Immunogenicity of HIV Subtype C DNA and MF59-Adjuvanted Subtype C Envelope Protein

**DOI:** 10.1093/cid/ciz1239

**Published:** 2020-01-04

**Authors:** Mina C Hosseinipour, Craig Innes, Sarita Naidoo, Philipp Mann, Julia Hutter, Gita Ramjee, Modulakgotla Sebe, Lucas Maganga, Michael E Herce, Allan C deCamp, Kyle Marshall, One Dintwe, Erica Andersen-Nissen, Georgia D Tomaras, Nonhlanhla Mkhize, Lynn Morris, Ryan Jensen, Maurine D Miner, Giuseppe Pantaleo, Song Ding, Olivier Van Der Meeren, Susan W Barnett, M Juliana McElrath, Lawrence Corey, James G Kublin, HVTN 111 Protocol Team, Nicole Frahm, Barbara Metch, Marguerite Koutsoukos, Stewart Reid, Bupe Sichalwe, Mah Asombang, Christine Namakobo, Sam Mundia, Lumbwe Banda, Joyce Mapanza, Jacinta Shilimi, Emmanuel Kapesa, Abisai Kisinda, Cornelia Lueer, Lilian Njovu, Wiston William, Faith Mlagalila, Elizabeth Ntapara, Willhelmina Olomi, Nnhamo Chiwerengo, Revocatus Kunambi, Bahati Myombe, Rosemary Mwilinga, Neema Mbinda, Joyce Masala, Joseph Mapunda, On Ho, Denelle Reilly, Liz Briesemeister, Marianne Hansen, Jill Zeller, Simba Takuva, Caroline Brackett, Jack Heptinstall, Kelly Seaton, David Beaumont, Lu Zhang, Sheetal Sawant, Marcella Sarzotti-Kelsoe, Tandile Hermanus, Valerie Bekker, Stephen De Rosa, Saleha Omarjee, Stephany Wilcox, Shamiska Rohith, Asiphe Basethi, Renaldo Noble, Daryl Morris

**Affiliations:** 1 University of North Carolina at Chapel Hill, Chapel Hill, NC, USA; 2 UNC Project-Malawi, Lilongwe, Malawi; 3 Aurum Institute, Klerksdorp, South Africa; 4 HIV Prevention Research Unit, South African Medical Research Council, Durban, South Africa; 5 Vaccine and Infectious Disease Division, Fred Hutchinson Cancer Research Center, Seattle, Washington, USA; 6 Division of AIDS, National Institute of Allergy and Infectious Diseases, National Institutes of Health, Bethesda, Maryland, USA; 7 Aurum Institute, Tembisa, South Africa; 8 NIMR–Mbeya Medical Research Center, Mbeya, Tanzania; 9 Centre for Infectious Disease Research in Zambia, Lusaka, Zambia; 10 Cape Town HVTN Immunology Laboratory, Cape Town, South Africa; 11 Duke Human Vaccine Institute, Duke University School of Medicine, Durham, North Carolina, USA; 12 National Institute for Communicable Diseases, National Health Laboratory Service, Johannesburg, South Africa; 13 Division of Immunology and Allergy, Centre Hospitalier Universitaire Vaudois, University of Lausanne, Lausanne, Switzerland; 14 EuroVacc Foundation, Lausanne, Switzerland; 15 GSK Vaccines, Rixensart, Belgium; 16 GSK Vaccines, Cambridge, Massachusetts, USA

**Keywords:** HIV vaccine, DNA prime/protein boost, subtype C, Biojector

## Abstract

**Background:**

The Pox-Protein Public-Private Partnership is performing a suite of trials to evaluate the bivalent subtype C envelope protein (TV1.C and 1086.C glycoprotein 120) vaccine in the context of different adjuvants and priming agents for human immunodeficiency virus (HIV) type 1 (HIV-1) prevention.

**Methods:**

In the HIV Vaccine Trials Network 111 trial, we compared the safety and immunogenicity of DNA prime followed by DNA/protein boost with DNA/protein coadministration injected intramuscularly via either needle/syringe or a needle-free injection device (Biojector). One hundred thirty-two healthy, HIV-1–uninfected adults were enrolled from Zambia, South Africa, and Tanzania and were randomized to 1 of 6 arms: DNA prime, protein boost by needle/syringe; DNA and protein coadministration by needle/syringe; placebo by needle/syringe; DNA prime, protein boost with DNA given by Biojector; DNA and protein coadministration with DNA given by Biojector; and placebo by Biojector.

**Results:**

All vaccinations were safe and well tolerated. DNA and protein coadministration was associated with increased HIV-1 V1/V2 antibody response rate, a known correlate of decreased HIV-1 infection risk. DNA administration by Biojector elicited significantly higher CD4^+^ T-cell response rates to HIV envelope protein than administration by needle/syringe in the prime/boost regimen (85.7% vs 55.6%; *P* = .02), but not in the coadministration regimen (43.3% vs 48.3%; *P* = .61).

**Conclusions:**

Both the prime/boost and coadministration regimens are safe and may be promising for advancement into efficacy trials depending on whether cellular or humoral responses are desired.

**Clinical Trials Registration:**

South African National Clinical Trials Registry (application 3947; Department of Health [DoH] no. DOH-27–0715–4917) and ClinicalTrials.gov (NCT02997969).

## Teaser

DNA and protein coadministration was associated with increased human immunodeficiency virus (HIV) type 1 V1/V2 antibody response. Biojector DNA administration elicited significantly greater CD4^+^ T-cell responses to HIV envelope protein than needle/syringe in the prime/boost but not the coadministration regimen.

Despite progress in human immunodeficiency virus (HIV) treatment, an estimated 1.8 million individuals were infected with HIV in 2017 [1], highlighting the need for an effective HIV vaccine. The RV144 vaccine trial is to date the only HIV vaccine trial that has demonstrated any efficacy [2]. The Pox-Protein Public-Private Partnership (P5) was established to improve on RV144 to create a more efficacious and durable vaccine in the predominately subtype C region of sub-Saharan Africa [3], where half the world’s HIV-infected population (36.9 million) resides [1]. The RV144 regimen, designed to protect against subtype B/E HIV type 1 (HIV-1) strains, was reformulated for the subtype C virus [4] and has demonstrated adequate immunogenicity [5]. Comprised of subtype C-adapted ALVAC and bivalent glycoprotein (gp) 120 protein with MF59 adjuvant (gp120/MF59), this vaccine regimen is currently under efficacy evaluation in South Africa (HIV Vaccine Trials Network [HVTN] 702). In parallel, the P5 correlates program is conducting several phase 1/2a trials designed to provide insights into potentially superior vaccine candidates and/or regimens based on favorable immune profiles. These phase 1/2a trials evaluate alternative strategies, adjuvants, and products for vaccine delivery, using common immunological end points.

HVTN 049 compared a DNA prime/gp140 protein boost regimen with a protein-only regimen for inducing humoral and cellular responses to HIV antigens [6]. The prime/boost strategy resulted in higher levels of envelope (Env) binding antibodies (bAbs) and homologous neutralizing antibodies (nAbs), greater CD4^+^ T-cell responses to Env antigens, and greater polyfunctionality of CD4^+^ T-cell responses. In nonhuman primate and mouse models, coadministration of DNA/protein elicited more robust humoral immunity than DNA alone or a prime/boost strategy [7, 8].

The most common vaccine administration method is via needle and syringe (needle/syringe). An alternative to needle/syringe is the Biojector, a needle-free injection system that results in improved interaction with antigen-presenting cells and enhanced immunological responses [9, 10]. The first of the P5 correlates program trials, HVTN 111, was designed to determine whether DNA vaccine administration method and schedule influence immune responses. Specifically, HVTN 111 evaluated the safety and immunogenicity of subtype C DNA-HIV-PT123 administered via needle/syringe or Biojector, and either boosted or coadministered with bivalent subtype C gp120/MF59 in HIV-uninfected, healthy adults in sub-Saharan Africa.

## METHODS

### Study Design

HVTN 111 was a phase 1 randomized, placebo-controlled study with 6 arms according to administration method (needle/syringe or Biojector) and prime/boost versus coadministration approaches. Participants were randomized to 1 of 6 regimens, according to the schema in Table 1. The DNA vaccine was administered via needle/syringe or Biojector, according to the regimen; adjuvanted protein vaccinations were administered via needle/syringe. When coadministered, the DNA and protein vaccines were administered contralaterally. The administration schedule in prime/boost via needle/syringe and prime/boost via Biojector matched the primary regimens of RV144 and HVTN 702. Coadministration via needle/syringe and coadministration via Biojector received 3 coadministrations of the 2 products, following a classic month 0-1-6 schedule (tetanus, diphtheria, hepatitis B). This approach is based on the observation that peak humoral immunogenicity is reached after 3 doses of protein, with no additional benefit of a fourth dose [11], and dampening of immunoglobulinin (Ig) G3 responses with additional protein administrations (unpublished data). The longer rest between the second and third vaccinations is preferred for antibody maturation.

**Table 1.** HIV Vaccine Trials Network 111 Study Schema

Group	Participants, No.	Vaccination Month			
		0	1	3	6
Prime/boost (S)	30	DNA-HIV-PT123 (S)	DNA-HIV-PT123 (S)	DNA-HIV-PT123 (S) + gp120/MF59 (S)	DNA-HIV-PT123 (S) + gp120/MF59 (S)
Coadministration (S)	30	DNA-HIV-PT123 (S) + gp120/MF59 (S)	DNA-HIV-PT123 (S) + gp120/MF59 (S)	Placebo (S) + Placebo (S)	DNA-HIV-PT123 (S) + gp120/MF59 (S)
Placebo (S)	6	Placebo (S)	Placebo (S)	Placebo (S)	Placebo (S)
Prime/boost (B)	30	DNA-HIV-PT123 (B)	DNA-HIV-PT123 (B)	DNA-HIV-PT123 (B) + gp120/MF59 (S)	DNA-HIV-PT123 (B) + gp120/MF59 (S)
Coadministration (B)	30	DNA-HIV-PT123 (B) + gp120/MF59 (S)	DNA-HIV-PT123 (B) + gp120/MF59 (S)	Placebo (B) + Placebo (S)	DNA-HIV-PT123 (B) + gp120/MF59 (S)
Placebo (B)	6	Placebo (B)	Placebo (B)	Placebo (B)	Placebo (B)

Abbreviations: B, Biojector; gp, glycoprotein; HIV, human immunodeficiency virus; S, needle/syringe.

### Study Participants

HVTN 111 enrolled 132 healthy, HIV-negative adults, aged 18–40 years, assessed as low risk for HIV infection, who agreed to study requirements and provided written informed consent in their preferred language. Good general health was determined by medical history, physical examination, and laboratory tests, including hematology, chemistry, and hepatitis serology. All female participants agreed to consistent contraception; pregnant and breastfeeding women were excluded. Eligibility criteria are described in Supplementary Table 1.

Participants were enrolled at 1 site in Zambia (Matero), 3 sites in South Africa (Isipingo, Klerksdorp, and Tembisa), and 1 site in Tanzania (Mbeya). HVTN 111 was approved by the research ethics committee of the participating sites. The study was registered with the South African National Clinical Trials Registry (application 3947; Department of Health [DoH] no. DOH-27–0715–4917) and ClinicalTrials.gov (NCT02997969).

### Study Products

The DNA vaccine, designated DNA-HIV-PT123 (IPPOX Foundation), comprises a mixture of 3 DNA plasmids in a 1:1:1 ratio, each at 1.33 mg: (1) subtype C ZM96 *gag*, (2) subtype C ZM96 *gp140*, and (3) subtype C CN54 *pol-nef*. The DNA vaccine was delivered at a total dose of 4 mg. The bivalent subtype C gp120 Env protein vaccine comprises subtype C TV1.C gp120 Env and subtype C 1086.C gp120 Env (GlaxoSmithKline Biologicals), each at a dose of 100 µg. The protein vaccine was mixed with MF59 adjuvant (Seqirus). The placebo was 0.9% sodium chloride for injection.

### Study Procedures

Participants were followed up for 12 months after the initial vaccination with safety evaluations and procedures as per Supplementary Methods. Adverse events (AEs) were reported over 30 days after each vaccination visit, with a subset of AEs being reported for the duration of the study.

### Immunogenicity Assays

All laboratory assays were performed blinded to treatment group with validated and qualified methods published elsewhere. Measurements included bAb, nAb and T-cell responses at the peak immunogenicity time point (2 weeks after final vaccination). A list of the specific antigens used in all immunogenicity assays is found in Supplementary Table 2.

### bAb Multiplex Assay

HIV-1–specific IgG bAb responses were measured at 1:50 dilution by an HIV-1 bAb multiplex assay against specific HIV-1 antigens, including vaccine-matched subtype C 96ZM651.C gp140, and V1V2 antigens 1086.C V1V2 and CaseA2_gp70_V1V2.B, as described elsewhere [12–15].

### nAb Assays

Neutralizing activity was measured against HIV Env-pseudotyped viruses as a function of reductions in Tat-regulated luciferase reporter gene expression in TZM-bl cells. Neutralization titers were measured against subtype C vaccine-matched strains TV1c8.2.C (tier 1A), 96ZM651.C (tier 2), Ce1086_B2.C (tier 2), and subtype C MW965.26.C (tier 1A) and judged positive if the neutralization titer was >10, as described elsewhere [16] (Supplementary Methods).

### Intracellular Cytokine Staining Assay

Peripheral blood mononuclear cells, collected at baseline and the peak immunogenicity time point, were isolated and cryopreserved from whole blood, as described elsewhere [17]. T-cell responses to vaccine-matched antigens were measured by intracellular cytokine staining (ICS), as described elsewhere [18, 19] (Supplementary Methods). The 17-color ICS panel is described in Supplementary Table 3.

### Statistical Analysis

#### Randomization

The randomization allocation sequence was obtained by computer-generated random numbers and provided to each site through the HVTN statistics and data monitoring center’s Web-based randomization system.

#### Positivity Calls and Response Comparisons

Barnard exact and Wilcoxon rank sum tests were used to compare the response rates and magnitudes for responders, respectively, between 2 groups [20]. Two-sided 95% confidence intervals for binomial proportions were calculated using the Wilson score method [21]. All tests were 2 sided with no adjustment for multiple comparisons, and differences were considered statistically significant at *P* <.05. For positivity analyses of ICS and bAb multiplex assays, see the Supplementary Methods.

#### T-cell Polyfunctionality Analyses

Combinatorial Polyfunctionality Analysis of Single Cells (COMPASS) analysis was used to analyze antigen-specific T-cell subsets [22]. The functionality score (FS) is defined as the estimated proportion of antigen-specific subsets detected among all possible ones. The polyfunctionality score (PFS) is similar but weighs the different subsets by their degree of functionality, naturally favoring subsets with higher degrees of functions, motivated by the observation that higher-degree function has been correlated with good outcomes in certain vaccine studies (Supplementary Methods).

## RESULTS

### Study Population and Schema

**Figure 1.**
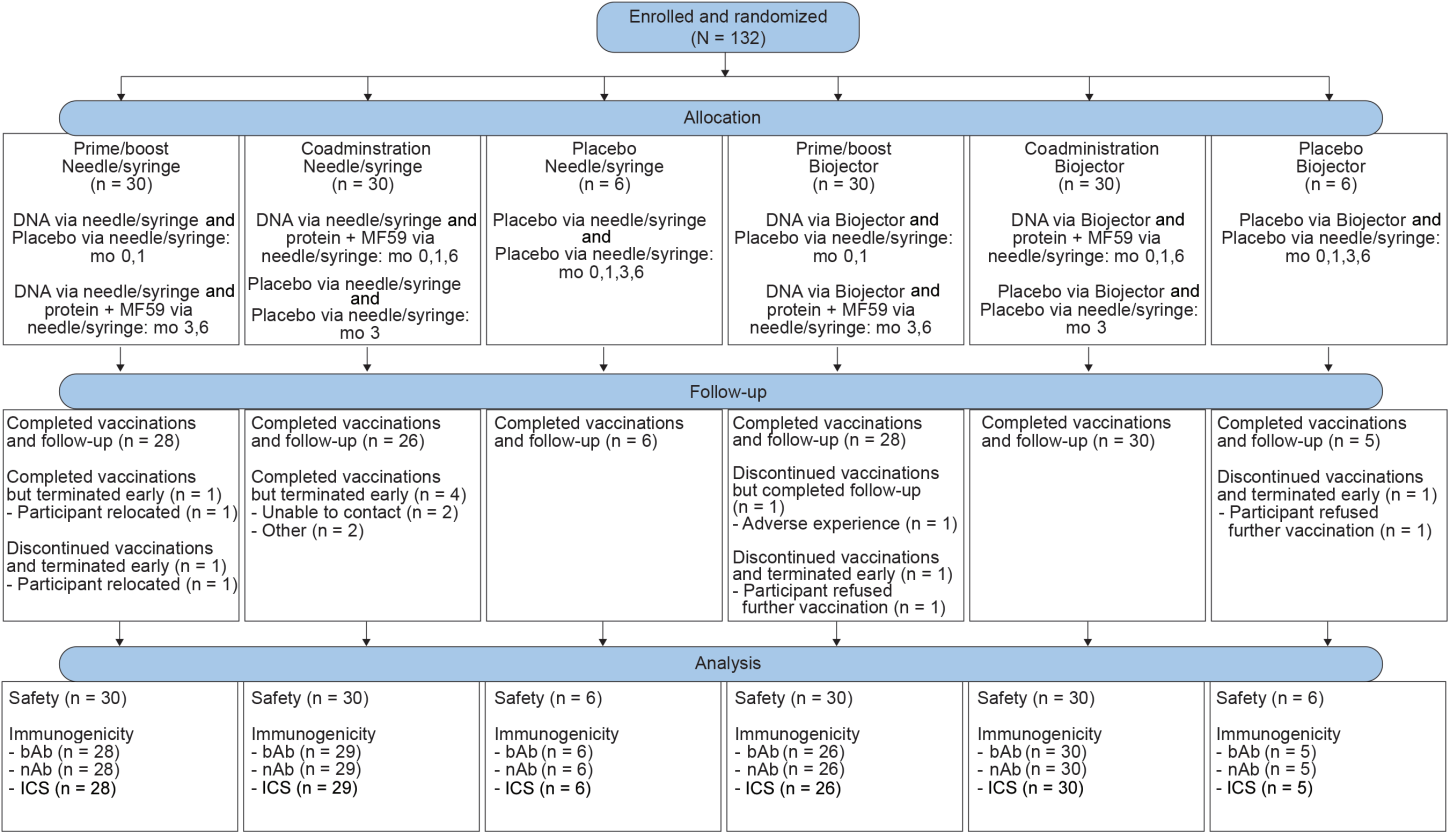
HIV Vaccine Trials Network (HVTN) 111 CONSORT diagram, showing enrollment and follow-up of participants in HVTN 111, including availability of samples for immunological testing. Abbreviations: bAb, binding antibody; CONSORT, consolidated standards of reporting trials; HIV, human immunodeficiency virus; ICS, intracellular cytokine staining; nAb, neutralizing antibody.

One hundred thirty-two healthy, HIV-uninfected, low-risk participants were enrolled at 5 sites between 21 June 2016 and 13 July 2017 (Table 2). Thirty participants were allocated to each of 4 vaccine arms and 6 participants to each placebo arm. Overall, 123 of 132 participants (93%) completed all vaccinations and follow-up (Figure 1).

**Table 2.** Baseline Demographic Characteristics and Vaccination Frequencies of Participants Enrolled in HIV Vaccine Trials Network 111, According to Randomization Arm

Characteristic or Vaccination Frequency	Participants, No. (%)^a^						
	Needle/Syringe			Biojector			
	Prime/Boost (n = 30)	Coadministration (n = 30)	Placebo (n = 6)	Prime/Boost (n = 30)	Coadministration (n = 30)	Placebo (n = 6)	Total (N = 132)
Sex							
Male	12 (40)	10 (33)	3 (50)	17 (57)	19 (63)	5 (83)	66 (50)
Female	18 (60)	20 (67)	3 (50)	13 (43)	11 (37)	1 (17)	66 (50)
Race							
Black	30 (100)	30 (100)	6 (100)	29 (97)	30 (100)	6 (100)	131 (99)
Asian	0 (0)	0 (0)	0 (0)	0 (0)	0 (0)	0 (0)	0 (0)
Indian	0 (0)	0 (0)	0 (0)	0 (0)	0 (0)	0 (0)	0 (0)
Mixed	0 (0)	0 (0)	0 (0)	1 (3)	0 (0)	0 (0)	1 (1)
Other	0 (0)	0 (0)	0 (0)	0 (0)	0 (0)	0 (0)	0 (0)
Age group							
18–20 y	7 (23)	8 (27)	0 (0)	9 (30)	7 (23)	4 (67)	35 (27)
21–30 y	19 (63)	20 (67)	5 (83)	16 (53)	21 (70)	2 (33)	83 (63)
31–40 y	4 (13)	2 (7)	1 (17)	5 (17)	2 (7)	0 (0)	14 (11)
Age, median (range), y	23.0 (19–39)	24.0 (18–38)	25.5 (21–38)	24.0 (18–37)	22.0 (18–37)	20.0 (18–27)	24.0 (18–39)
Vaccination frequencies							
mo 0	30 (100)	30 (100)	6 (100)	30 (100)	30 (100)	6 (100)	132 (100)
mo 1	29 (97)	30 (100)	6 (100)	27 (90)	30 (100)	5 (83)	127 (96)
mo 3	29 (97)	30 (100)	6 (100)	28 (93)	29 (97)	5 (83)	127 (96)
mo 6	29 (97)	30 (100)	6 (100)	28 (93)	30 (100)	5 (83)	128 (97)

Abbreviation: HIV, human immunodeficiency virus.^a^Data represent no. (%) of participants unless otherwise specified.

### Safety and Tolerability

**Figure 2.**
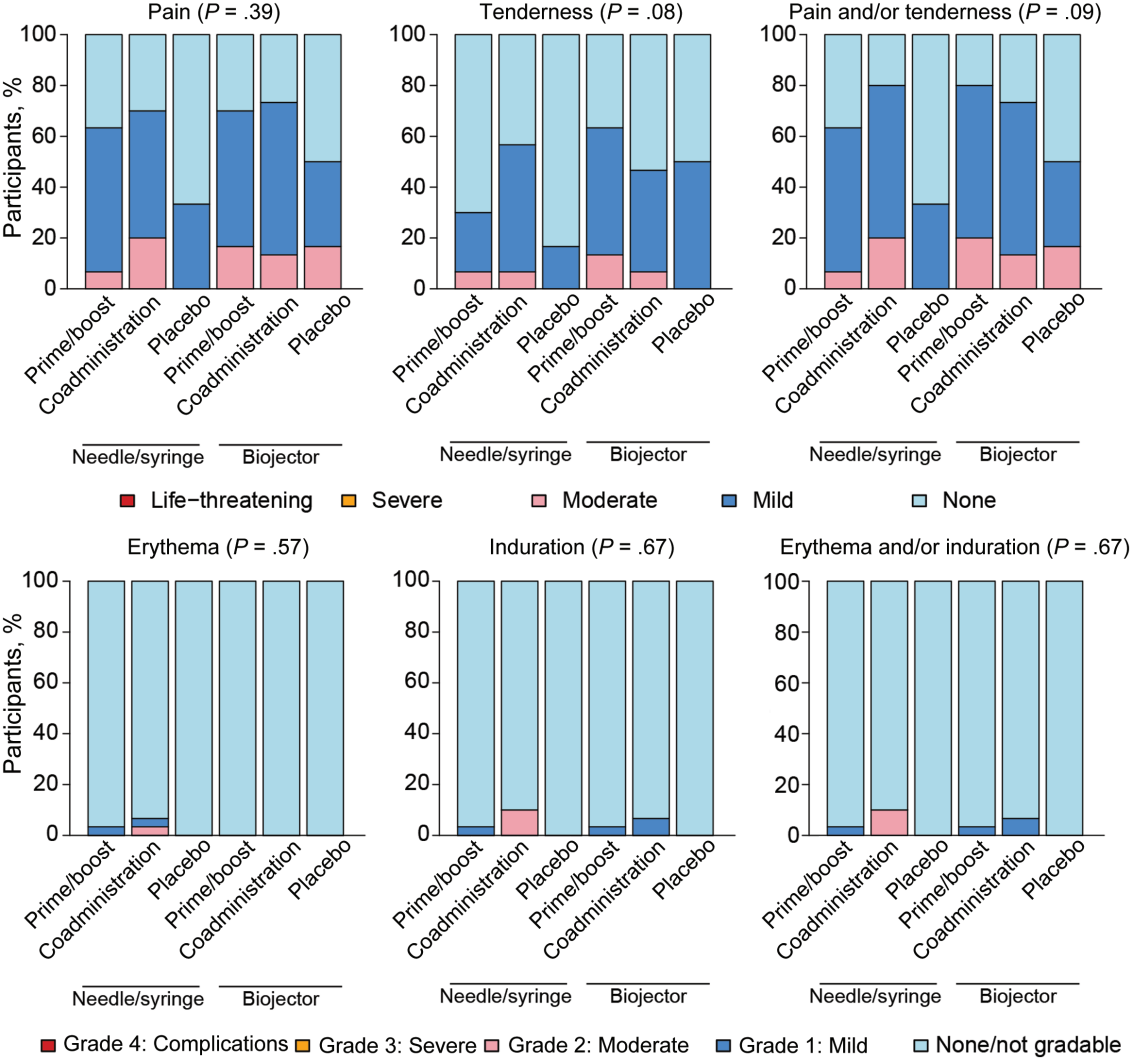
Reactogenicity in HIV Vaccine Trials Network (HVTN) 111. Local reactogenicity symptoms according to treatment arm and severity grade. Abbreviation: HIV, human immunodeficiency virus.

All 132 participants received the first vaccinations, 127 received the second and third vaccinations, and 128 received the fourth vaccinations. All vaccinations were well tolerated. No related serious AEs, related severe AEs, or severe local reactogenicity symptoms were reported. Apart from 1 case of grade 3 elevated temperature, no severe systemic reactogenicity symptoms were reported (Supplementary Figure 1) No significant differences were observed in local and systemic reactogenicity symptoms between vaccine and placebo recipients, overall. When the DNA and protein injections were analyzed separately, pain and/or tenderness were more common in some of the vaccine groups when compared with placebo for DNA injections (Supplementary Figure 2); the same was true for protein injections. Pain, tenderness, and pain and/or tenderness were more common with Biojector than needle/syringe administration. For DNA injections via needle/syringe, the median severity grade was “none,” whereas for DNA injections via Biojector, the median severity grade was “mild” (Figure 2).

Four participants discontinued receiving vaccinations, 3 because of relocation or refusal, and 1 because of an undisclosed pre-existing condition of diabetes. Overall, 81 AEs were reported; 78 of them mild or moderate. One grade 3 AE for type 1 diabetes mellitus, 1 grade 4 AE for increased aspartate aminotransferase, and 1 grade 4 serious AE for intentional self-injury were reported, all deemed not related to study product. Two AEs were deemed related to study product, both mild in severity: 1 case of decreased neutrophil count and 1 case of malaise. One participant reported injection site blistering at the site of DNA administration via Biojector. Blistering occurred after the second, third, and fourth vaccinations, and resolved spontaneously.

### Humoral Responses

**Figure 3.**
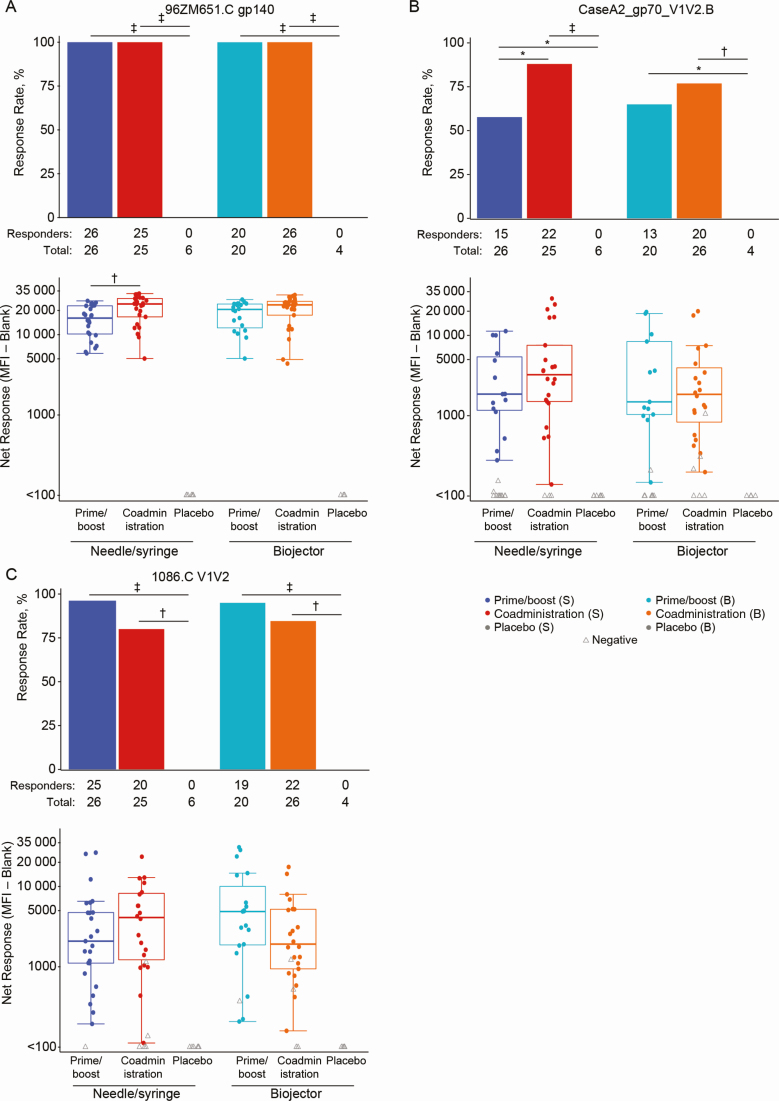
Binding antibody responses. BAMA response rates (bar charts) and magnitudes (box plots) by treatment arm for the following antigens: 96ZM651.C glycoprotein (gp) 140 (*A*), CaseA2_gp70_V1V2.B (*B*), and 1086.C V1V2 (*C*). Bar charts show positive response rates. Box plots show responses and are based on positive responders only (shown as colored circles); negative responders are shown as gray triangles. *P ≤ .05; †*P* ≤ .01; ‡*P* ≤ .001. Abbreviations: B, Biojector; BAMA, binding-antibody multiplex assay; MFI, mean fluorescence intensity; S, needle/syringe.

In all vaccine recipients (100%) IgG responses developed to vaccine-matched Env gp120/gp140 (1086.C gp120, TV1c8.2.C gp120, 96ZM651.C gp140) and the group M consensus gp120/gp140 (Con 6 gp120, Con S gp140 CFI) at high magnitudes (Figure 3 and data not shown). As expected, no positive responses were seen among placebo recipients. Responses to vaccine-matched 96ZM651.C gp140 were significantly higher in the coadministration group versus prime/boost group via needle/syringe (*P* = .007) (Figure 3A); there were no significant differences between other treatment groups. The magnitude of the IgG response to both subtype B and C V1V2 antigens, known correlates of decreased HIV-1 risk in RV144 [12, 15], trended higher in the coadministration group versus prime/boost group via needle/syringe (Figure 3B and 3C).

**Figure 4.**
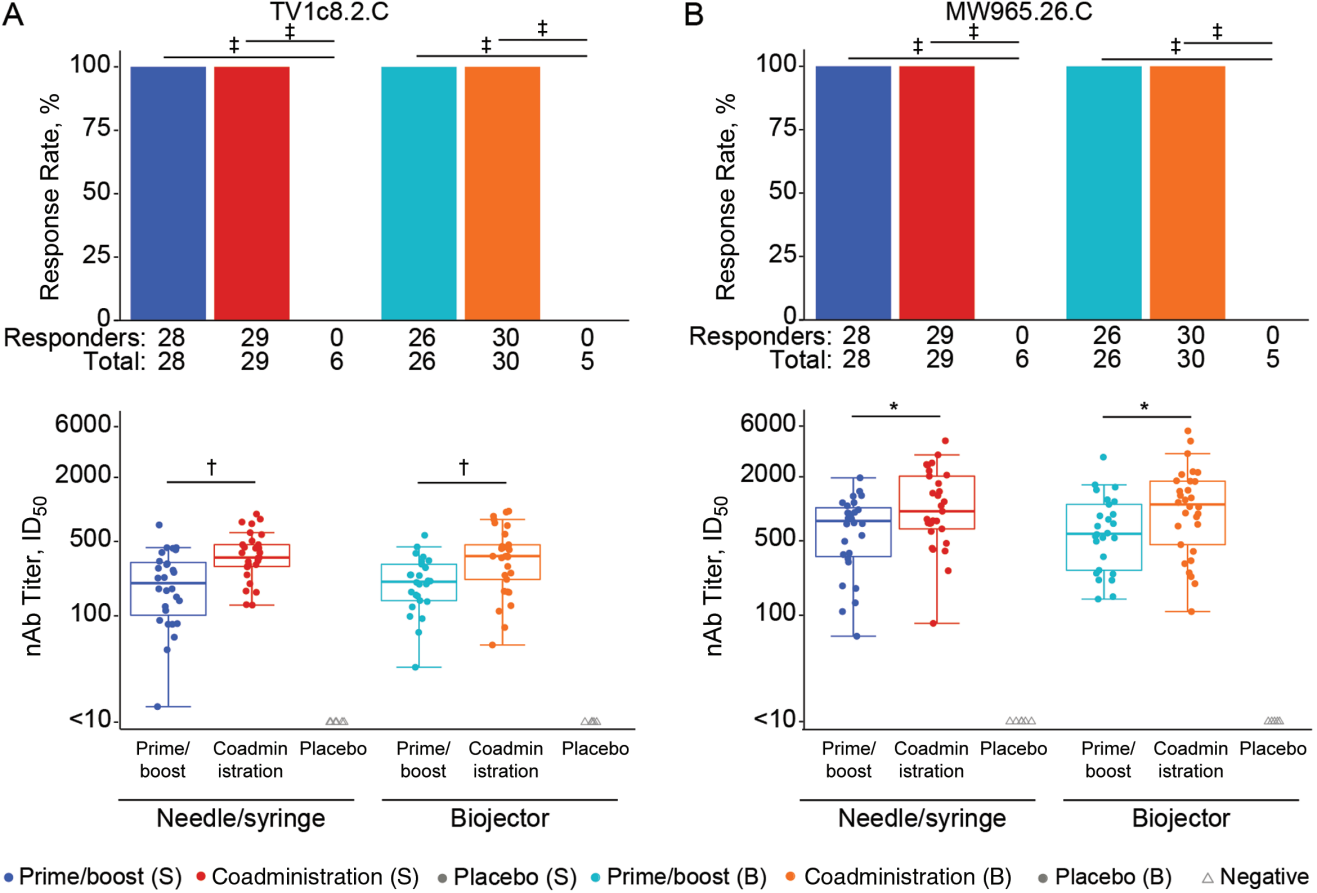
Neutralizing antibody (nAb) responses. Response rates (bar charts) and nAb titers (box plots) against TV1c8.2.C (*A*) and MW965.26.C (*B*) are shown by treatment arm. Bar charts show positive response rates. Box plots show responses and are based on positive responders only (shown as colored circles); negative responders are shown as gray triangles. *P ≤ .05; †*P* ≤ .01; ‡*P* ≤ .001. Abbreviations: B, Biojector; ID50, 50% infectious dose; S, needle/syringe.

All vaccine recipients and no placebo recipients developed nAb responses against tier 1A strains TV1c8.2.C and MW965.26.C (Figure 4). There were no positive responders against tier 2 vaccine strains in any group (data not shown). nAb responses in the coadministration via needle/syringe group were significantly higher in magnitude than those in the prime/boost via need/syringe group for TV1c8.2.C (*P* = .0004) (Figure 4A) and MW965.26.C (*P* = .03) (Figure 4B). The same pattern emerged in the Biojector groups between coadministration and prime/boost: TV1c8.2.C (*P* = .002) and MW965.26.C (*P* = .03). There were no significant differences between nAb response magnitudes for TV1c8.2.C or MW965.26.C for vaccine groups of the same regimen but with different methods of administration.

### T-cell Responses

**Figure 5.**
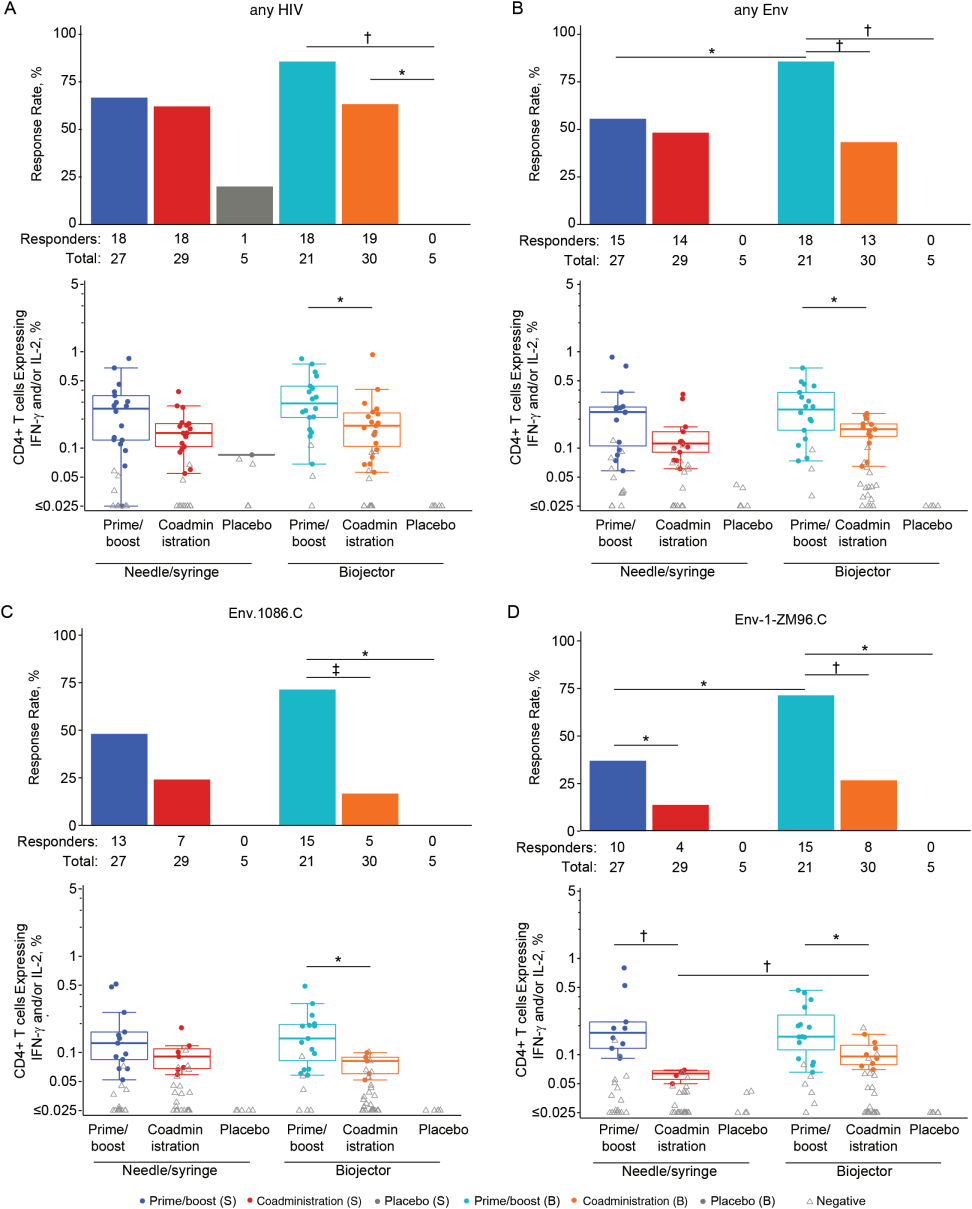
CD4^+^ T-cell responses, as measured by intracellular cytokine staining. Response rate (bar charts) and magnitude (box plots) 2 weeks after the final vaccination by treatment arm are shown for the following vaccine-matched peptide pools: any human immunodeficiency virus (HIV) (*A*), Any envelope (Env) (*B*), Env.1086.C (*C*), and Env-1-ZM96.C (*D*). Any HIV is the sum of any Pol, any Env, Nef-CN54, and Gag-ZM96.C, where any Pol is the sum of Pol-1-CN54 and Pol-2-CN54. Any Env is the maximum of Env ZM96, Env.1086.C, and Env.TV1.C, where Env ZM96 is the sum of Env-1-ZM96.C and Env-2-ZM96.C. Bar charts show positive response rates. Box plots show responses and are based on positive responders only (shown as colored circles); negative responders are shown as gray triangles. *P ≤ .05; †*P* ≤ .01; ‡*P* ≤ .001. Abbreviations: B, Biojector; IFN, interferon; IL-2, interleukin; S, needle/syringe; Pol, polyfunctionality.

The vaccine regimens induced HIV-specific CD4^+^ T cells expressing interleukin 2 (IL-2) and/or interferon (IFN) γ in most vaccine recipients (Figure 5). Response magnitudes did not differ significantly between prime/boost regimens to the combined vaccine-matched peptides (“any HIV”) (Figure 5A), combined vaccine-matched Env peptides (“any Env”) (Figure 5B) and individual vaccine-matched Env peptides (Figure 5C and 5D). Response rates and magnitudes did not differ significantly overall between prime/boost and coadministration via needle/syringe (Figure 5A–5C), with the exception of higher responses to Env-1-ZM96.C for prime/boost (*P* = .047 for response rate; *P* = .002 for magnitude) (Figure 5D). The prime/boost regimen via Biojector produced significantly higher CD4^+^ T-cell response rates than coadministration for all Env antigens tested: any Env (*P* = .002), Env.1086.C (*P* < .0001), Env-1-ZM96.C (*P* = .002), Env-2-ZM96.C (*P* = .002), and Env.TV1.C (*P* = .02) (Figure 5B–5D). Response magnitudes for prime/boost were also significantly higher than coadministration via Biojector for any HIV (*P* = .01), any Env (*P* = .02), Env.1086.C (*P* = .04), and Env-1-ZM96.C (*P* = .047) (Figure 5).

Comparing prime/boost regimens administered via different methods, Biojector elicited significantly higher CD4^+^ T-cell response rates to any Env than needle/syringe (85.7% vs 55.6%, respectively; *P* = .02) (Figure 5B). There was no significant difference in coadministration regimens between Biojector and needle/syringe for any Env (Figure 5B). Participants who received the coadministration via Biojector had significantly higher response magnitudes to Env-1-ZM96.C than those who received coadministration via needle/syringe (*P* = .004) (Figure 5D); there were no other significant differences between these 2 groups for the other antigens tested.

CD8^+^ T-cell responses were infrequently induced in all groups (Supplementary Figure 3) with no statistically significant differences in response rates or magnitudes with needle/syringe or Biojector administration. When the prime/boost regimens were compared with each other, Biojector resulted in significantly higher response rates than needle/syringe for any HIV (33.3% vs 7.4%, respectively; *P* = .02) and Gag-ZM96.C (16.7% vs 0.0%; *P* = .03). There were no significant differences in CD8^+^ T-cell response rates between coadministration vaccines via the 2 administration methods, nor were there any differences in response magnitudes between prime/boost vaccinees (via needle/syringe or Biojector) or coadministration vaccinees (via needle/syringe or Biojector).

**Figure 6.**
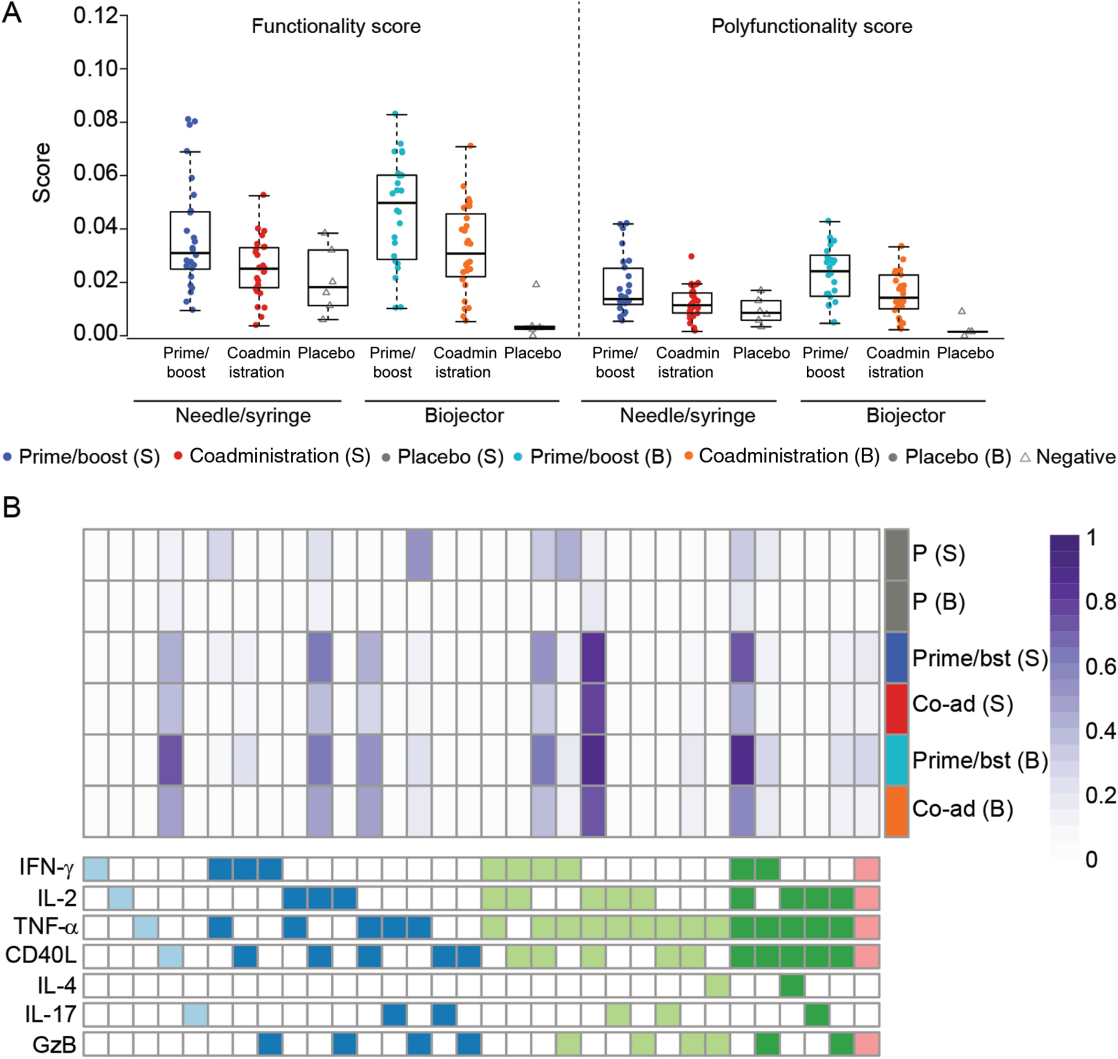
Functionality and polyfunctionality according to study arm. *A,* Functionality and polyfunctionality scores of CD4^+^ T-cell subsets to any envelope (Env) ZM96 2 weeks after the final vaccination. To determine scores for Env ZM96, data for Env-1-ZM96.C and Env-2-ZM96.C were combined before fitting the Combinatorial Polyfunctionality Analysis of Single Cells (COMPASS) model. *B,* Heat map of mean COMPASS posterior probabilities for CD4^+^ T-cell responses to any Env ZM96 among vaccine and placebo recipients. Rows correspond to mean posterior probabilities of participants in each treatment group. Each cell shows the probability (ranging from white [0] to purple [1]) that the corresponding cell subset (*column*) demonstrates an antigen-specific response in the corresponding treatment group (*row*). Abbreviations: B, Biojector; Co-ad, coadministration; GzB, granzyme B; IFN, interferon; IL-2, IL-4, and IL-17, interleukin 2, 4, and 17; P, Placebo; Prime/bst, prime/boost; S, needle/syringe; TNF, tumor necrosis factor.

We performed a polyfunctionality analysis to assess IFN-γ, IL-2, tumor necrosis factor (TNF) α, CD40L, interleukin 4, interleukin 17, and granzyme B coexpression using COMPASS analysis [22]. Comparisons of FS and PFS between prime/boost and coadministration via needle/syringe revealed significant differences only for the combined vaccine-matched Env ZM96 (“any Env ZM96”) (*P* = .04 for FS) and the Env-1-ZM96.C (*P* = .03 for FS and *P* = .02 for PFS), with prime/boost via needle/syringe having higher FS and PFS (Figure 6A). When prime/boost was compared with coadministration delivered by Biojector, FS and PFS were significantly higher in the prime/boost group for any Env ZM96 (*P* < .001 for FS and *P* = .09 for PFS) (Figure 6A) and for each of the individual Env peptide pools (Env.1086.C [*P* = .005 for FS and *P* = .004 for PFS], Env-1-ZM96.C [*P* = .008 and *P* = .01, respectively] and Env-2-ZM96.C [both *P* = .02]; data not shown). There were no significant differences in FS and PFS comparisons by administration route.

In Figure 6B, a heat map of the COMPASS posterior probabilities allows visualization of the different populations contributing to the FS. All vaccine regimens induced polyfunctional CD4^+^ T cells expressing 2, 3, and 4 functional combinations of IFN-γ, IL-2, TNF-α, and CD40L. Triple functional CD4^+^ T cells coexpressing IL-2, TNF-α, and CD40L or IFN-γ, TNF-α, and CD40L were the 2 dominant populations responding to any Env-ZM96 in all vaccine groups, regardless of administration method. In contrast, the 4-function subset expressing IFN-γ IL-2, TNF-α, and CD40L was more likely to be expressed in the prime/boost regimens. CD40L single-expressing cells were most common in the prime/boost group vaccinated via Biojector.

### Vaccine-induced Seroreactivity

Vaccine-induced seroreactivity, assessed by means of commercial HIV serological methods, occurred in 1 vaccine recipient (0.8%). This individual was in the coadministration via Biojector group and tested reactive only with the Alere Determine HIV-1/2 Ag/Ab Combo test.

## DISCUSSION

In the first reported clinical trial from the P5 correlates program, HVTN 111, the subtype C DNA vaccine (DNA-HIV-PT123) and the bivalent subtype C gp120/MF59 were generally safe and well tolerated in healthy volunteers in sub-Saharan Africa. Humoral responses were robust in all vaccination arms. Some cellular responses were enhanced with the prime/boost approach compared with the coadministration regimen when administered via Biojector. Given that humoral responses were higher after coadministration and cellular responses higher after prime/boosting, the potential use of these approaches may depend on whether humoral or cellular responses are more desirable. Notably, despite the lower CD4^+^ T-cell responses in the coadministration arms, the potent humoral responses indicate that T cells are sufficiently stimulated by vaccination to support antibody responses.

Local pain and/or tenderness were more common with administration via Biojector than with administration via needle/syringe, albeit these reactions were mostly mild. One participant experienced self-limiting blistering at the injection site after Biojector administration, something that has been reported elsewhere at a low frequency [9]. Vaccinations with the bivalent subtype C gp120/MF59 were also safe and well tolerated.

The HVTN 111 candidate vaccine regimens elicited robust humoral responses in response rate and magnitude. The antibody response magnitude increased with coadministration of the protein and DNA components. The coadministration arms received 3 gp120/MF59 dosages compared with 2 in the prime/boost approach. One objective of this study was to determine whether the frequency and magnitude of the V1V2 IgG correlate of decreased HIV-1 risk [12, 14, 15] could be differentially modulated. We observed that the response magnitude did trend higher in the coadministration arm compared with the prime/boost arm. Consistent with expectations that Biojector administration largely influences cellular responses, we saw no difference in humoral responses with the different administration methods.

The prime/boost approach elicited higher cellular response rates than the coadministration regimen. The prime/boost strategy provides 1 additional DNA vaccine administration compared with the coadministration regimen, which may explain some of the observed differences. With respect to administration method, the Biojector enhanced CD4^+^ T-cell response rate and magnitude when compared to standard vaccination via needle/syringe, but only in the prime/boost regimen. These CD4^+^ T-cell response rates exceed those seen when the canarypox vector ALVAC is given with the same protein boost (Moodie Z et al, submitted).

The current study presented no safety concerns, which supports advancement of the suite of P5 vaccines. However, Biojector administration requires the Biojector device, carbon dioxide cartridges, and specific syringes that are not widely available and will require a more complicated supply chain management plan in a public health setting than needle/syringe strategies. The modest gains in cellular responses may be of questionable added value given the necessary complexities for implementation [12, 15]. Coadministration may induce earlier protective humoral responses. A public health advantage to coadministration would include simplicity in dosing, with the same vaccine (DNA and protein) administered at each vaccination time point. This ease of use would need to be balanced with potential reduction in the cellular responses seen with the standard prime/boost approach.

Importantly, both approaches were performed with the same bivalent subtype C proteins used in the ongoing efficacy trial HVTN 702. HVTN 111 represents the first time these particular proteins have been assessed with a different priming agent, and in different administration regimens. In this study, we confirmed the differentiated skewing of the humoral and cellular responses in the prime/boost and coadministration regimens. The results of this trial help pave the road to potential refinements of the HVTN 702 regimen with the aim to fine-tune the immunological responses deemed most critical for vaccine efficacy based on correlates of risk/protection. Ideally these targeted refinements can help shape a future vaccine to enhance levels of efficacy beyond those seen in RV144. Thus, this study sets the foundation for optimizing vaccine regimens based on observed correlates of risk/protection from existing HIV-1 efficacy trials, in concert with upcoming efficacy trial results.

## Supplementary Data

Supplementary materials are available at *Clinical Infectious Diseases* online. Consisting of data provided by the authors to benefit the reader, the posted materials are not copyedited and are the sole responsibility of the authors, so questions or comments should be addressed to the corresponding author.

ciz1239_suppl_Supplementary_Figure_1

ciz1239_suppl_Supplementary_Figure_2

ciz1239_suppl_Supplementary_Figure_3

ciz1239_suppl_Supplementary_Material

## Notes

***Acknowledgments.*** The authors thank all study participants enrolled in HIV Vaccine Trials Network (HVTN) 111. The authors gratefully acknowledge the participation and support of many colleagues and staff on the HVTN 111 protocol team. The authors thank Nicole Frahm, Barbara Metch, and Marguerite Koutsoukos for their contributions to this study. At the clinical research sites, the authors thank Stewart Reid, Bupe Sichalwe, Mah Asombang, Christine Namakobo, Sam Mundia, Lumbwe Banda, Joyce Mapanza, Jacinta Shilimi, and Bertha Chibwe at the Lusaka-Matero site and Emmanuel Kapesa, Abisai Kisinda, Cornelia Lueer, Lilian Njovu, Wiston William, Faith Mlagalila, Elizabeth Ntapara, Willhelmina Olomi, Nnhamo Chiwerengo, Revocatus Kunambi, Bahati Myombe, Rosemary Mwilinga, Neema Mbinda, Joyce Masala, and Joseph Mapunda at the NIMR–Mbeya Medical Research Center. The authors thank On Ho, Denelle Reilly, Liz Briesemeister, Marianne Hansen, Jill Zeller, and Simba Takuva for their roles in protocol development and study implementation. The authors also thank Caroline Brackett, Jack Heptinstall, and Kelly Seaton for the binding antibody assays; David Beaumont, Lu Zhang, Sheetal Sawant, and Sahill Patel for data management; Marcella Sarzotti-Kelsoe and quality assurance unit for quality assurance oversight; and Tandile Hermanus and Valerie Bekker for the neutralizing antibody assays. For the intracellular cytokine staining assays, the authors thank Stephen De Rosa, Saleha Omarjee, Stephany Wilcox, Shamiska Rohith, Asiphe Basethi, and Renaldo Noble for conducting the laboratory assays and Daryl Morris for performing Combinatorial Polyfunctionality Analysis of Single Cells (COMPASS) analysis. The authors thank Anthony Williams for data processing and quality control, April Randhawa for developing the assay plans and assistance with data management, and Ashley Clayton for assistance with manuscript preparation.

***Financial support***. This work was supported by NIAID, NIH (grants UM1 AI068614 [Leadership and Operations Center: HVTN], UM1 AI068618 [Laboratory Center: HVTN], UM1 AI068635 [Statistical and Data Management Center: HVTN], UM1 AI069453 [Klerksdorp Clinical Research Site], UM1 AI069422 [Durban-Isipingo Clinical Research Site], UM1 AI108568 [Mbeya Clinical Research Site], and UM1 AI069423 [University of North Carolina at Chapel Hill Global Clinical Trials Unit, Malawi Clinical Research Site]; grants HHSN272201300033C/HHSN272201600012C for development of the glycoprotein [gp] 120 envelope proteins TV1.C and 1086.C and support for the manufacture and release of the gp120 clinical grade material to Novartis Vaccines and Diagnostics [now GlaxoSmithKline (GSK)]); the Bill & Melinda Gates Foundation (OPP1110830; funding to the Fred Hutchinson Cancer Research Center to support the implementation of HVTN 111 at the Klerksdorp Clinical Research Site, Lusaka-Matero Clinical Research Site, and Tembisa Clinical Research Site; and global health grant OPP1017604 to Novartis Vaccines and Diagnostics [now GSK]). The DNA vaccine was provided by the IPPOX Foundation, Switzerland, through support from the Collaboration of AIDS Vaccine Discovery of the Bill & Melinda Gates Foundation (grant OPP52845).

***Potential conflicts of interest.*** M. C. H., S. N., G. R., L. Maganga, O. D., E. A. N., G. D. T., N. M., and L. Morris report grants from the NIAID/NIH, during the conduct of the study. P. M., A. C. D., K. M., R. J., M. D. M., M. J. M., L. C., and J. G. K. report grants from NIAID/NIH and the Bill & Melinda Gates Foundation, during the conduct of the study. C. I. reports grants from NIAID/NIH and funding from Fred Hutchinson Cancer Research Center via a subaward agreement, during the conduct of the study. M. S. and M. E. H. report funding from the Fred Hutchinson Cancer Research Center via a subaward agreement, during the conduct of the study. G. P. and S. D. report grants from the Bill & Melinda Gates Foundation, during the conduct of the study. O. V. D. M. and S. W. B., on behalf of GSK (formerly Novartis Vaccines and Diagnostics), report grants from NIAID/NIH and the Bill & Melinda Gates Foundation, during the conduct of the study. During the study, O. V. D. M. and S. W. B. were full-time employees of GSK and held stocks and shares with GSK. Furthermore, S. W. B. has a patent issued for the HIV vaccine composition. J. H. is a full-time paid employee of the NIAID. M. C. H., C. I., S. N., P. M., G. R., L. Maganga, A. C. D., K. M., O. D., E. A. N., N. M., L. Morris, R. J., M. D. M., O. V. D. M., S. W. B., M. J. M., L. C., and J. G. K. are recipients of NIAID funding, and this report is a result of activities funded by NIAID. G. D. T. reports grants from GSK, outside the submitted work, and has patents for HIV immunogens and incidence assays pending. J. H. was not involved with the process of funding these awards, nor in their administration or scientific aspects, and, in accordance with NIAID policies, is deferred from decisions regarding funding of coauthors for a requisite period. Within the terms of the grant award of the cooperative agreement with the HVTN, J. H. served as the NIAID medical officer of the HVTN 111 trial and contributed to the study design, safety reviews and monitoring, and review of the data and manuscript. No pharmaceutical company or other agency paid J. H. for contributions to this study or manuscript. All authors have submitted the ICMJE Form for Disclosure of Potential Conflicts of Interest. Conflicts that the editors consider relevant to the content of the manuscript have been disclosed.
