# Supplementary figures and images for: Phase 1 Human Immunodeficiency Virus (HIV) Vaccine Trial to Evaluate the Safety and Immunogenicity of HIV Subtype C DNA and MF59-Adjuvanted Subtype C Envelope Protein
Source: Clin Infect Dis. 2020 Jan 4;72(1):50–60. doi: 10.1093/cid/ciz1239 (PMC7823071; doi:10.1093/cid/ciz1239)

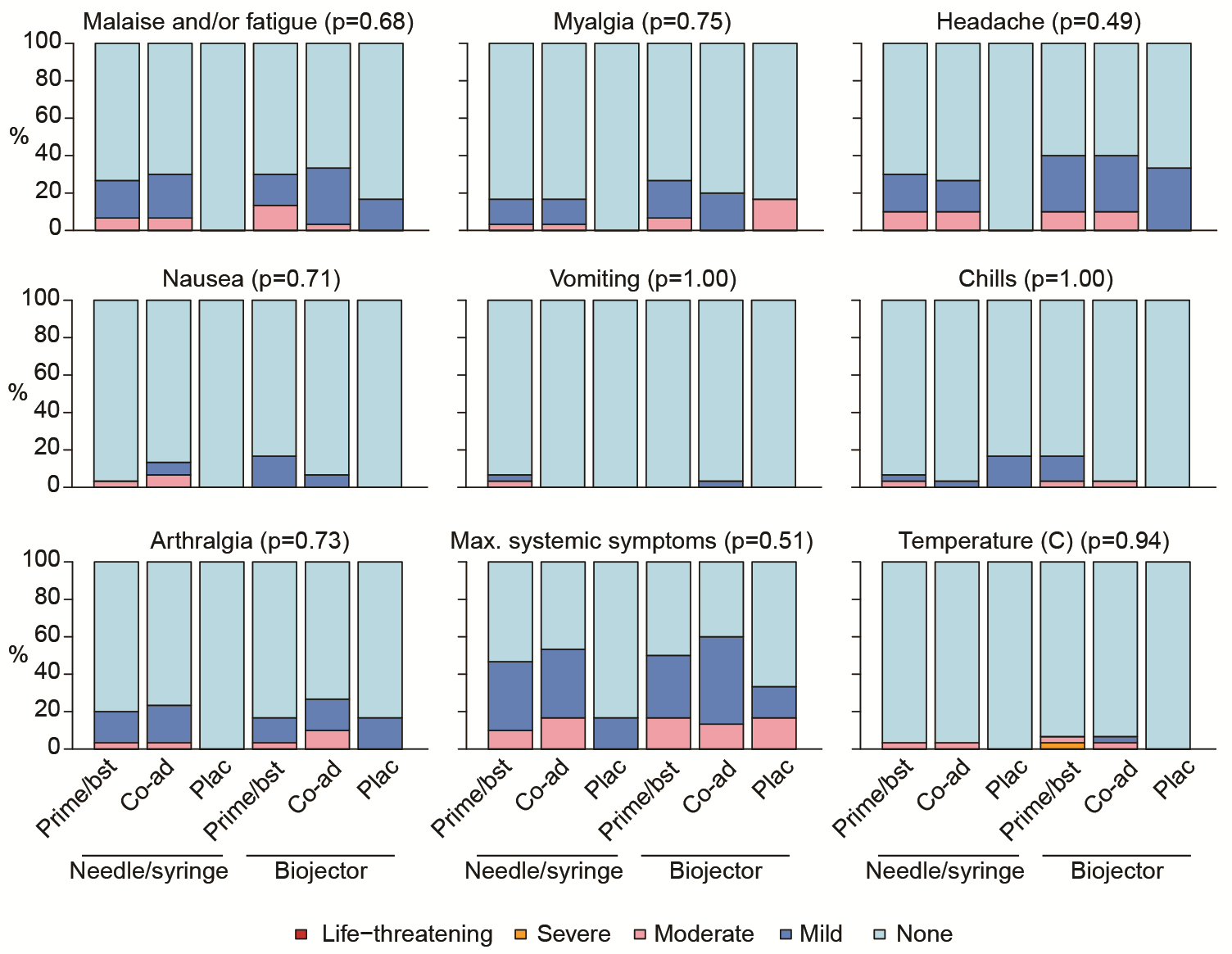

Supplement: ciz1239_suppl_Supplementary_Figure_1 [file ciz1239_suppl_supplementary_figure_1.png]

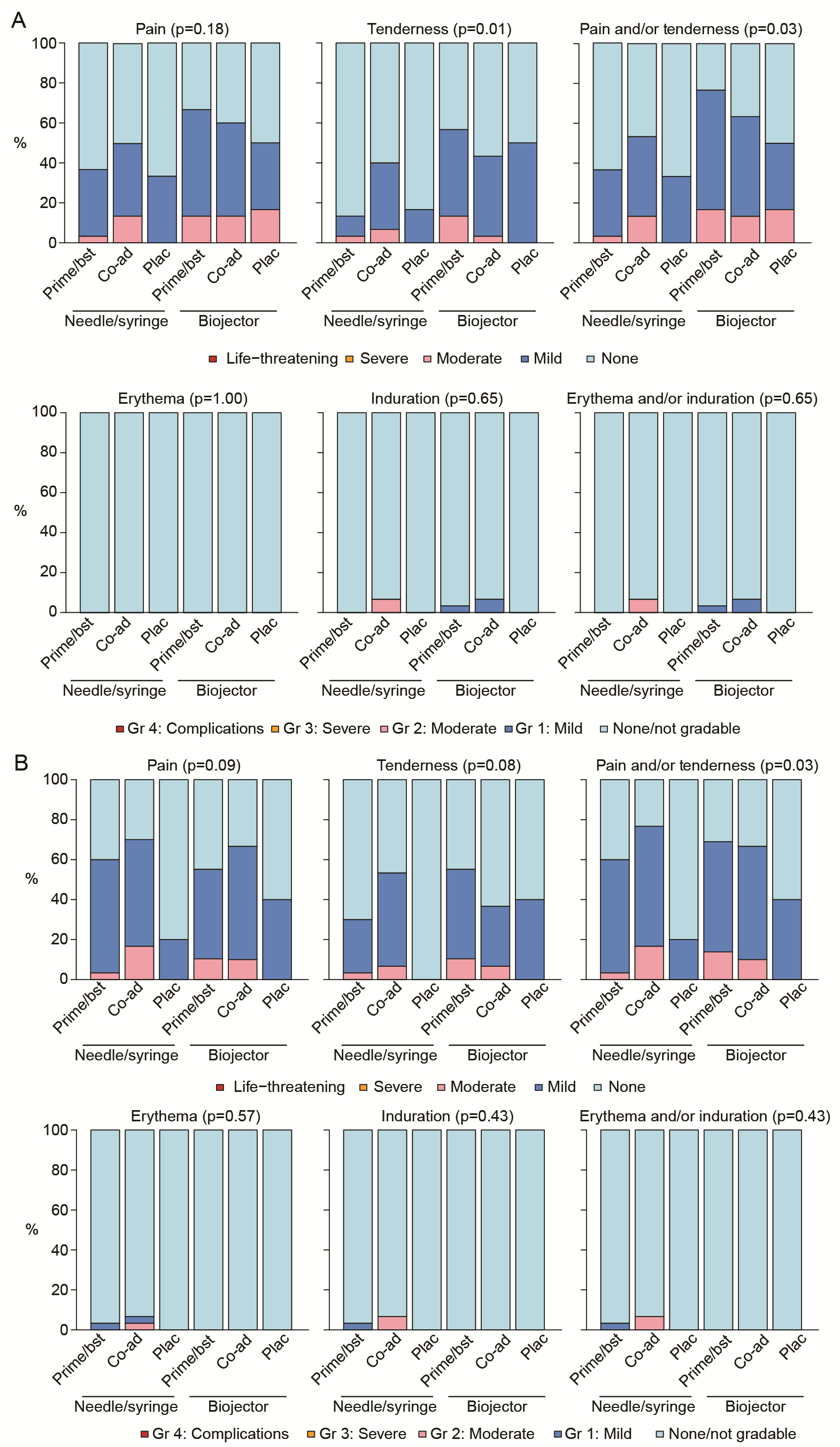

Supplement: ciz1239_suppl_Supplementary_Figure_2 [file ciz1239_suppl_supplementary_figure_2.png]

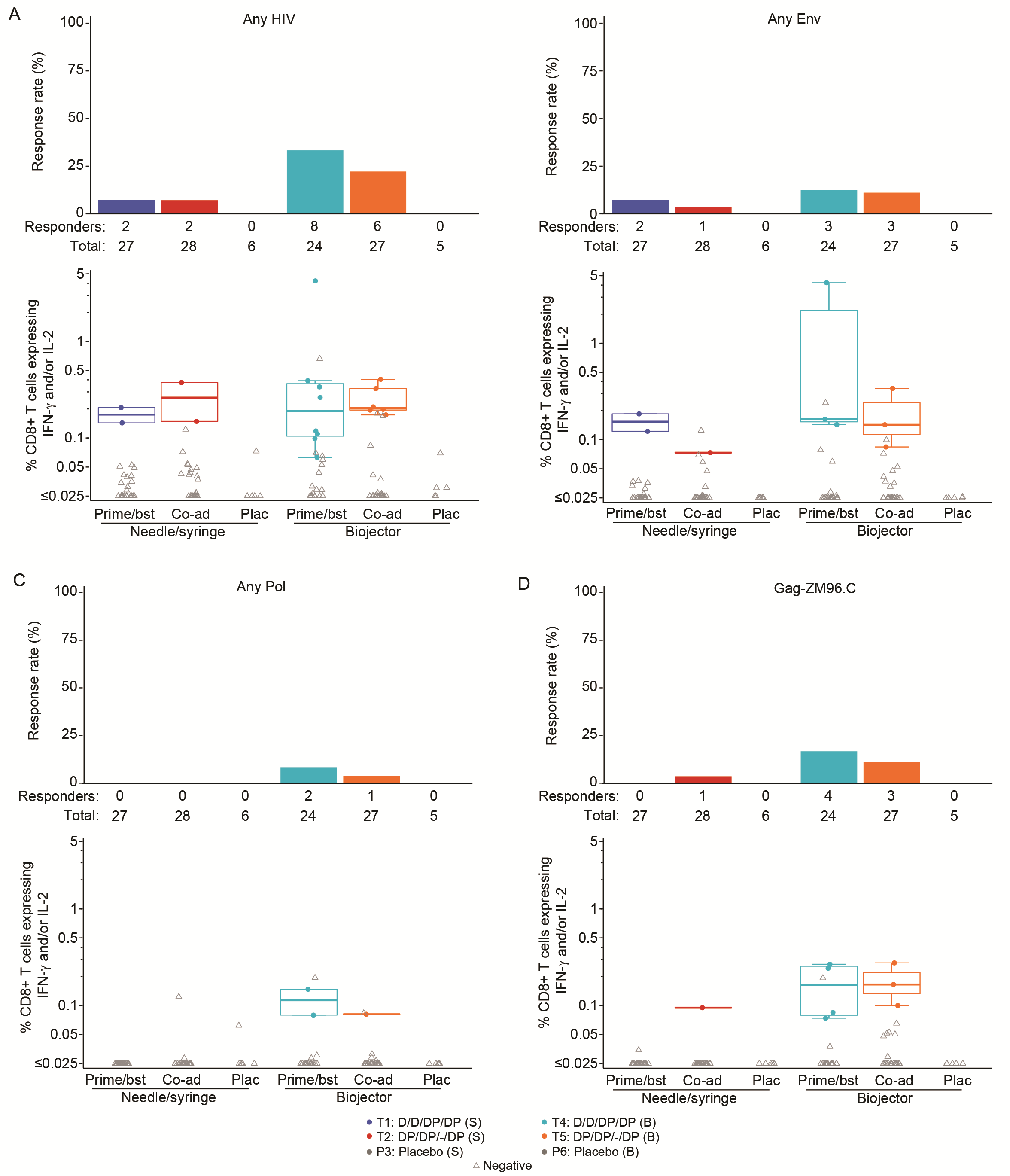

Supplement: ciz1239_suppl_Supplementary_Figure_3 [file ciz1239_suppl_supplementary_figure_3.png]
