# Supplementary material for: Phase 1 Human Immunodeficiency Virus (HIV) Vaccine Trial to Evaluate the Safety and Immunogenicity of HIV Subtype C DNA and MF59-Adjuvanted Subtype C Envelope Protein
Source: Clin Infect Dis. 2020 Jan 4;72(1):50–60. doi: 10.1093/cid/ciz1239 (PMC7823071; doi:10.1093/cid/ciz1239)
Supplement: ciz1239_suppl_Supplementary_Material [file ciz1239_suppl_supplementary_material.docx]

Supplemental Materials for

Phase 1 HIV vaccine trial to evaluate the safety and immunogenicity of HIV subtype C DNA and MF59-adjuvanted subtype C Env protein

**Supplemental Methods**

*Study procedures*

Study visits occurred at Months 0, 0.25, 1, 1.5, 3, 3.5, 6, 6.25, 6.5, 9, and 12, with vaccinations occurring at Months 0, 1, 3, and 6. Safety evaluations included physical examinations and standard clinical chemistry and hematological tests, urine dipstick, as well as pregnancy tests for female participants. Local injection site (pain, tenderness, erythema, and induration) and systemic reactogenicity symptoms (increased body temperature, malaise and/or fatigue, myalgia, headache, chills, arthralgia, nausea, and vomiting) were assessed daily for seven days following each vaccination or until resolution. A subset of AEs being reported for the duration of the study (including serious AEs [SAEs], AEs of special interest, new chronic conditions requiring medical intervention for ≥30 days, STIs, and AEs leading to early participant withdrawal or early discontinuation of study product administration). The number and percentage of participants experiencing each type of reactogenicity sign or symptom was tabulated by maximum severity and treatment arm for Protocol Safety Review Team (PSRT) review.

Severity of reactogenicity and AEs were graded based on the Division of AIDS Table for Grading the Severity of Adult and Pediatric Adverse Events (DAIDS AE Grading Table), Version 1.0, December 2004 (Clarification dated August 2009).

Immunogenicity Assays

*Neutralizing antibody assays*

Titer was defined as the serum dilution that reduced relative luminescence units by 50% (ID_50_) compared with relative luminescence units in virus control wells (cells and virus only) after subtraction of background relative luminescence units (cells only). A response was judged positive if the neutralization titer was >10.

*Intracellular cytokine staining assay*

PBMCs were stimulated with either peptide pools (15-mer peptides overlapping by 11 amino acids) for the vaccine-matched proteins (Env-ZM96.C, Env.TV1.C, Env.1086.C, Gag-ZM96.C, Pol-CN54, Nef-CN54), dimethyl sulfoxide or Staphylococcus enterotoxin B in the presence of costimulatory antibodies and brefeldin A. Cells were incubated then stained with an antibody staining panel, acquired on a BD LSRFortessa flow cytometer, and analyzed using FlowJo version 9.9.4. Data were excluded from subsequent analyses if fewer than 5000 CD4+ or CD8+ T cells were acquired.

Statistical Analysis

*Sample size calculations*

At each site, the pharmacist with responsibility for dispensing study products maintained security of the treatment assignments. Participants and site staff (except for site pharmacists) were blinded to participant treatment arm assignment (e.g., vaccine or placebo) but not to the administration method (Biojector or needle/syringe). All randomized participants were included in the safety analyses. Kruskal-Wallis tests were used to determine if there were any significant differences in reactogenicity between the treatment regimens.

All randomized participants with reliable assay data based on blood draw dates within the allowable visit window were included in the immunogenicity analysis.

*Positivity calls and response comparisons*

For the binding antibody assays, samples were declared to have positive responses if they met three conditions: (1) the mean fluorescence intensity (MFI) minus blank values were ≥ antigen-specific threshold (based on the 95th percentile of the baseline visit serum samples and at least 100 MFI), (2) the MFI minus blank values were > 3 times the baseline (day 0) MFI minus blank values, and (3) the MFI values were > 3 times the baseline MFI values. “Blank” refers to a sample specific background value, measured using blank beads.

For the ICS assay, positivity for a peptide pool was based on comparing the percentage of T cells with positive staining for IL-2 and/ or IFN-γ between the experimental and negative control wells using a one-sided Fisher’s exact test with a p-value cutoff for positivity of 10^−5^, and with a Bonferroni multiplicity adjustment for the number of peptide pools [1, 2].

*T-cell polyfunctionality analyses*

For the COMPASS analysis, cell subsets that do not have at least 5 cells in at least 2 participants are excluded. Single functions for granzyme B and IL-4, and the dual combination of granzyme B with IL-4 were excluded from the analysis due to constitutive expression of granzyme B and high background for IL-4 for these particular combinations.

**Supplemental Tables**

**Supplemental Table 1.** **Inclusion and exclusion criteria for HVTN 111.**

| Inclusion criteria |
| --- |
| Age of 18 to 40 years |
| Access to a participating HVTN clinical research site |
| Ability and willingness to provide informed consent |
| Assessment of understanding |
| Agrees not to enroll in another study |
| Good general health |
| Willingness to receive HIV test results |
| Willingness to discuss HIV infection risks |
| Assessed by the clinic staff as being at “low risk” for HIV infection |
| Hemoglobin ≥ 11.0 g/dL (born female), ≥ 13.0 g/dL (born male) |
| White blood cell count = 3,300 to 12,000 cells/mm^3^ |
| Total lymphocyte count ≥ 800 cells/mm^3^ |
| Remaining differential either within institutional normal range or with site physician approval |
| Platelets = 125,000 to 550,000/mm^3^ |
| ALT, AST, and ALP < 1.25 times the institutional upper limit of normal |
| Creatinine ≤ institutional upper limit of normal |
| Negative HIV-1 and -2 blood test |
| Negative Hepatitis B surface antigen (HBsAg) |
| Negative anti-Hepatitis C virus antibodies (anti-HCV) |
| Negative urine glucose |
| Negative or trace urine protein |
| Negative or trace urine hemoglobin |
| Volunteers who were born female: negative serum or urine beta human chorionic gonadotropin (β-HCG) pregnancy test performed prior to vaccination on the day of initial vaccination |
| A volunteer who was born female must agree to consistently use effective contraception from at least 21 days prior to enrollment through the last required protocol clinic visit |
| Volunteers who were born female must also agree not to seek pregnancy through alternative methods |
|  |
| Exclusion criteria |
| Blood products received within 120 days |
| Investigational research agents received within 30 days |
| Body mass index (BMI) ≥ 40; or BMI ≥ 35 with 2 or more of the following: systolic blood pressure > 140 mm Hg, diastolic blood pressure > 90 mm Hg, current smoker, known hyperlipidemia |
| Intent to participate in another study |
| Pregnant or breastfeeding |
| HIV vaccine(s) received in a prior HIV vaccine trial |
| Non-HIV experimental vaccine(s) received within the last 5 years |
| Live attenuated vaccines other than influenza vaccine received within 30 days before first vaccination or scheduled within 14 days after injection |
| Influenza vaccine or any vaccines that are not live attenuated vaccines and were received within 14 days |
| Allergy treatment with antigen injections within 30 days before first vaccination or that are scheduled within 14 days after first vaccination |
| Immunosuppressive medications |
| Serious adverse reactions to vaccines or to vaccine components |
| Immunoglobulin received within 60 days |
| Autoimmune disease |
| Immunodeficiency |
| Untreated or incompletely treated syphilis infection |
| Clinically significant medical condition |
| Any medical, psychiatric, occupational, or other condition |
| Psychiatric condition that precludes compliance with the protocol |
| Current anti-tuberculosis (TB) prophylaxis or therapy |
| Asthma other than mild, well-controlled asthma |
| Diabetes mellitus type 1 or type 2 |
| Thyroidectomy, or thyroid disease |
| Hypertension |
| Bleeding disorder |
| Malignancy |
| Seizure disorder |
| Asplenia |
| History of hereditary angioedema, acquired angioedema, or idiopathic angioedema |
| ALT = alanine transaminase; AST = aspartate aminotransferase; ALP = alkaline phosphatase |

**Supplemental Table 2. Details of the BAMA, ICS, and nAb antigens, including HIV-1 viral strain information.**

| Assay | Antigen/  virus class | Full antigen/virus name | Antigen/virus name used in plots and/or throughout text | Viral strain information:  Subtype.Country.Year.Stage* |
| --- | --- | --- | --- | --- |
| BAMA | gp140 | 96ZM651.gp140C.avi | 96ZM651.C gp140 | C.ZM.96.6 |
|  | gp140 | Con S gp140 CFI | Con S gp140 CFI | [Group M Consensus] |
|  | gp120 | 1086C_D7gp120.avi/293F | 1086.C gp120 | C.MW.04.1-2 |
|  | gp120 | Con 6 gp120/B | Con 6 gp120 | [Group M Consensus] |
|  | gp120 | TV1c8_D11gp120.avi/293F | TV1c8.2.C gp120 | C.ZA.98.6 |
|  | V1V2 | gp70_B.CaseA_V1_V2 | CaseA2_gp70_V1V2.B | B.US.88.6 |
|  | V1V2 | C.1086_V1_V2 Tags | 1086.C V1V2 | C.MW.04.1-2 |
|  | V1V2 | gp70-TV1.GSKvacV1V2/293F | TV1c8.2.C V1V2 | C.ZA.98.6 |
|  | gp41 | gp41 | gp41 | B.xx.xx.xx |
| TZM-bl nAb | PSV** | TV1c8.2 | TV1c8.2.C | C.ZA.98.6 |
|  | PSV** | MW965.26 | MW965.26.C | C.MW.93.6 |
|  | PSV** | Ce1086_B2 | Ce1086_B2.C | C.MW.04.1-2 |
|  | PSV** | 96ZM651.02 | 96ZM651.C | C.ZM.96.6 |
| ICS*** |  | 1086 gp120 Env | Env.1086.C | C.MW.04.1-2 |
|  |  | Env-1-ZM96 | Env-1-ZM96.C | C.ZM.96.6 |
|  |  | Env-2-ZM96 | Env-2-ZM96.C | C.ZM.96.6 |
|  |  | Gag-ZM96 | Gag-ZM96.C | C.ZM.96.6 |
|  |  | TV1 gp120 Env | Env.TV1.C | C.ZA.98.6 |
|  |  | Pol-1-CN54 | Pol-1-CN54 | 07.CN.97.xx |
|  |  | Pol-2-CN54 | Pol-2-CN54 | 07.CN.97.xx |
|  |  | Nef-CN54 | Nef-CN54 | 07.CN.97.xx |
| * Subtype is denoted by a capital letter; country of origin is denoted by the 2 digit International Organization for Standardization code; year isolated is denoted by 2 digits; when country of origin and year isolated are unknown, they are denoted as “xx”; and stage is denoted by “a” (acute, if Fiebig stage is unknown) or “1”, “2”, “3”, “4”, “5”, or “6” (acute or early chronic, where the number or range corresponds to the Fiebig stage or range of stages when known)  **PSV = Env-pseudotyped virus  *** In plots and throughout text, Any Env = max((Env-1-ZM96.C + Env-2-ZM96.C), Env.1086.C, Env.TV1.C); Any Pol = Pol-1-CN54 + Pol-2-CN54; Any HIV = Any Env + Any Pol + Nef-CN54 + Gag-ZM96.C | | | | |

**Supplemental Table 3.** **Panel of antibodies used for ICS.**

|  | Specificity | Fluorochrome | Clone | Manufacturer | Catalogue number |
| --- | --- | --- | --- | --- | --- |
| Viability marker | AViD | NA | NA | Life Technologies | L34957 |
| Cellular surface markers | CCR7 | BV785 | G043H7 | BioLegend | 353229 |
|  | CD14 | BV510* | M5E2 | BioLegend | 301842 |
|  | CD56 | BV570 | HCD56 | BioLegend | 318330 |
|  | CD45RA | APC H7 | HI100 | BD Biosciences | 560674 |
|  | CXCR5 | PE-Dazzle594 | J252D4 | BioLegend | 356928 |
|  | ICOS (CD278) | BV711 | DX29 | BD Biosciences | 563833 |
|  | PD-1 (CD279) | BV605 | EH12.2H7 | BioLegend | 329924 |
| Intracellular markers | CD3 | BUV737 | UCHT1 | BD Biosciences | 564307 |
|  | CD4 | BUV395 | SK3 | BD Biosciences | 563550 |
|  | CD8 | BV650 | RPA-T8 | BD Biosciences | 563821 |
|  | CD154 | APC | TRAP-1 | BD Biosciences | 555702 |
|  | IFNγ | V450 | B27 | BD Biosciences | 560371 |
|  | Granzyme B | Alx700 | GB11 | BD Biosciences | 560213 |
|  | IL-2 | PE | MQ1-17H12 | BD Biosciences | 559334 |
|  | IL-4 | PerCP-Cy5.5 | MP4-25D2 | BioLegend | 500822 |
|  | IL-17a | PE-Cy7 | BL168 | BioLegend | 512315 |
|  | TNFα | FITC | MAb11 | eBioscience | 11-7349-82 |
| * CD14 and AViD are detected in the same channel | | | | | |

Supplemental Figures

Supplemental Figure 1. Systemic reactogenicity in HVTN 111. Systemic reactogenicity symptoms according to treatment arm and severity grade.


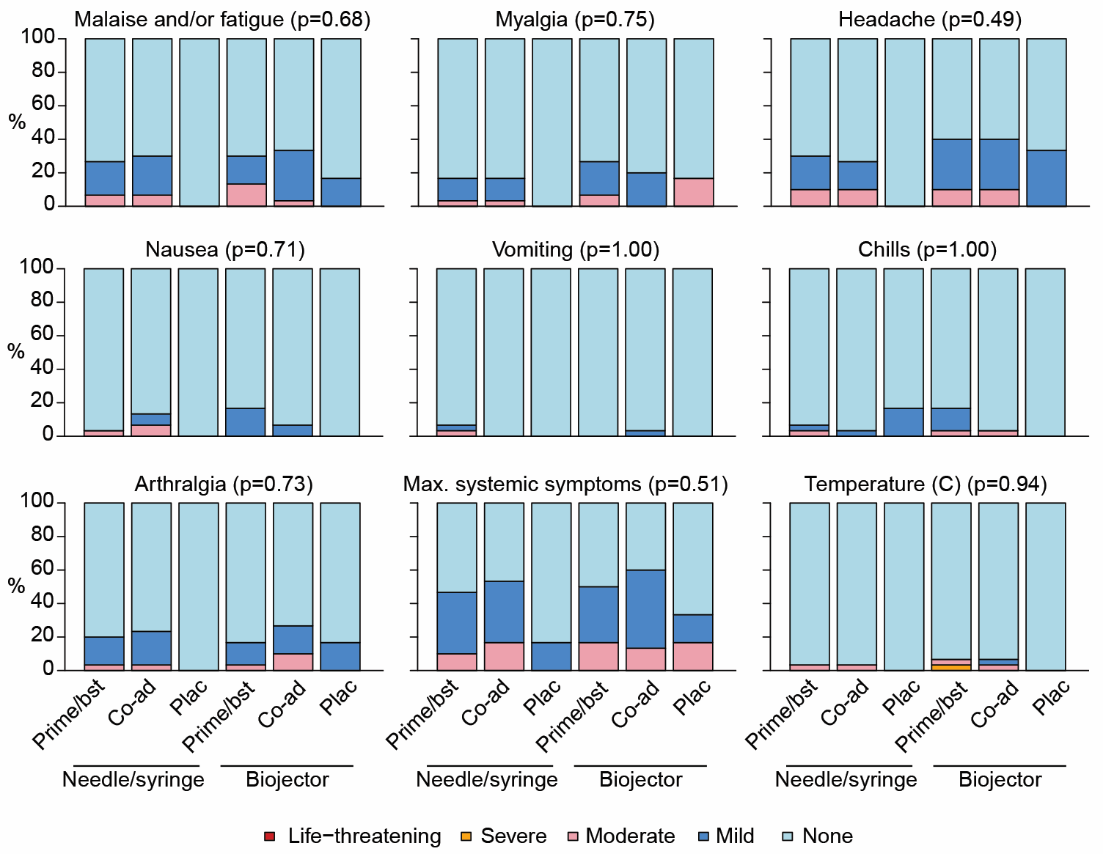


**
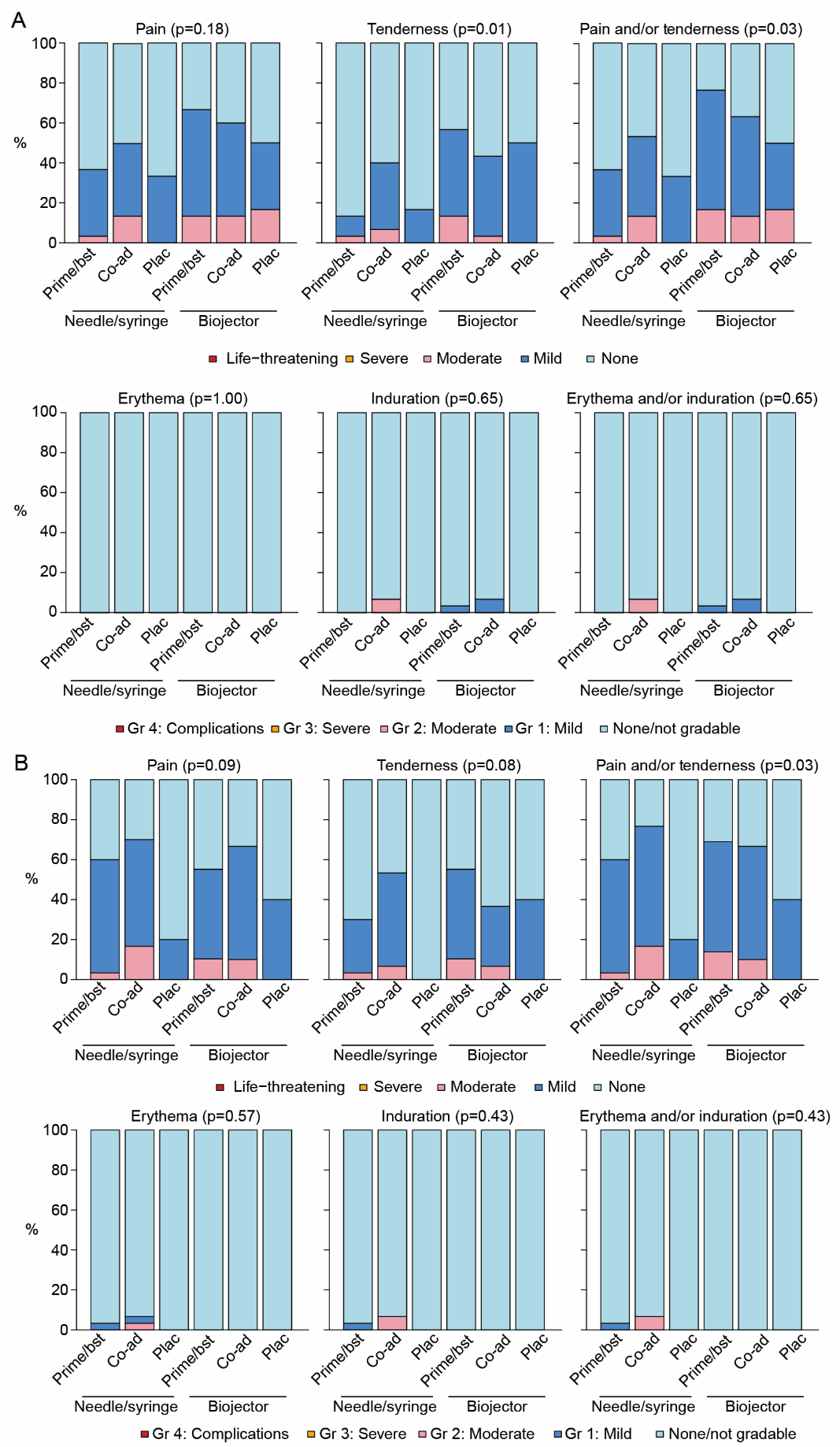
Supplemental Figure 2**. Local reactogenicity following (**A**) DNA injections and (**B**) protein injections.

**Supplemental Figure 3. CD8+ T-cell responses, as measured by ICS.** Response rate (bar charts) and magnitude (boxplots) by treatment arm for the following vaccine-matched peptide pools: (**A**) Any HIV; (**B**) Any Env; (**C**) Any Pol; (**D**) Gag-ZM96.C. Any HIV is the sum of Any Pol, Any Env, Nef-CN54, and Gag ZM96, Any Env is the maximum of Env ZM96, Env.1086.C, and Env.TV1.C, where Env ZM96 is the sum of Env-1-ZM96.C and Env-2-ZM96.C, and Any Pol is the sum of Pol-1-CN54 and Pol-2-CN54. Bar charts show positive response rates. Boxplots show responses and are based on positive responders only (shown as colored circles); negative responders are shown as grey triangles. D = DNA. DP = DNA and protein co-administration. - = placebo during the month 3 visit for T2 and T5. S = needle/syringe. B = Biojector.


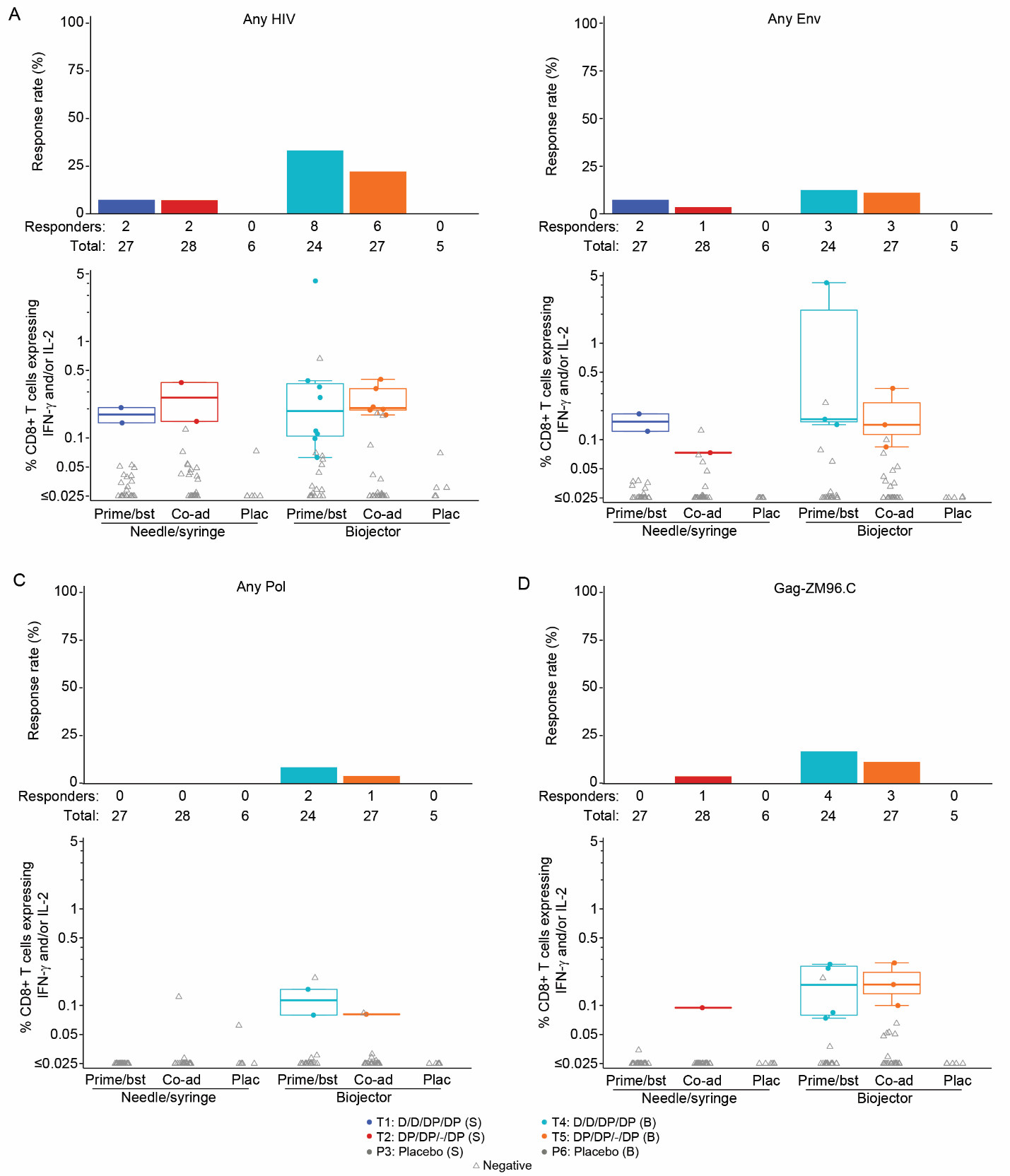


REFERENCES

1. Agresti A, Coull BA. Approximate Is Better than "Exact" for Interval Estimation of Binomial Proportions. The American Statistician 1998; 52(2): 119-26.

2. Horton H, Havenar-Daughton C, Lee D, et al. Induction of human immunodeficiency virus type 1 (HIV-1)-specific T-cell responses in HIV vaccine trial participants who subsequently acquire HIV-1 infection. J Virol 2006; 80(19): 9779-88.
